# Hybrid cardiac imaging using PET/MRI: a joint position statement by the European Society of Cardiovascular Radiology (ESCR) and the European Association of Nuclear Medicine (EANM)

**DOI:** 10.1007/s00330-017-5008-4

**Published:** 2018-05-02

**Authors:** Felix Nensa, Fabian Bamberg, Christoph Rischpler, Leon Menezes, Thorsten D. Poeppel, Christian la Fougère, Dietrich Beitzke, Sazan Rasul, Christian Loewe, Konstantin Nikolaou, Jan Bucerius, Andreas Kjaer, Matthias Gutberlet, Niek H. Prakken, Rozemarijn Vliegenthart, Riemer H. J. A. Slart, Stephan G. Nekolla, Martin L. Lassen, Bernd J. Pichler, Thomas Schlosser, Alexis Jacquier, Harald H. Quick, Michael Schäfers, Marcus Hacker, on behalf of the European Society of Cardiovascular Radiology (ESCR), Marco Francone, Jens Bremerich, Luigi Natale, Joachim Wildberger, Valentin Sinitsyn, the European Association of Nuclear Medicine (EANM) Cardiovascular Committee, Fabien Hyafil, Hein J. Verberne, Roberto Sciagrà, Alessia Gimelli, Christopher Übleis, Oliver Lindner

**Affiliations:** 1 Department of Diagnostic and Interventional Radiology and Neuroradiology, University Hospital Essen, Hufelandstrasse 55, 45147 Essen, Germany; 2 Department of Diagnostic and Interventional Radiology, University of Tuebingen, Hoppe-Seyler-Straße 3, 72076 Tübingen, Germany; 3 Department of Nuclear Medicine, Klinikum rechts der Isar, Technical University of Munich, Ismaninger Straße 22, 81675 Munich, Germany; 4 UCL Institute of Nuclear Medicine, and NIHR, University College London Hospitals Biomedical Research Centre, 5th Floor Tower, University College London Hospital, 235 Euston Road, London, NW1 2BU UK; 5 Klinik für Nuklearmedizin, Universitätsklinikum Essen, Hufelandstraße 55, 45122 Essen, Germany; 6 Nuklearmedizin und Klinische Molekulare Bildgebung, Otfried-Müller-Straße 14, 72076 Tübingen, Germany; 7 Department of Bioimaging and Image-Guided Therapy, Medical University Vienna, Währinger Gürtel 18-20, 1090 Vienna, Austria; 8 Department of Radiology and Nuclear Medicine, Medical University Vienna, Währinger Gürtel 18-20, Floor 5L, 1090 Vienna, Austria; 9 Maastricht Oncology Centre, Medical University Maastricht, P. Debyelaan 25, 6229 HX Maastrich, Netherlands; 10 Section of Endocrinology Research, University of Copenhagen, Panum Instituttet, Blegdamsvej 3, 2200, 12.3 Copenhagen N, Denmark; 11 Diagnostic and Interventional Radiology, University of Leipzig-Heart Center, Strümpellstrasse 39, 04289 Leipzig, Germany; 12 University Medical Center Groningen, Department of Radiology, University of Groningen, Hanzeplein 1, 9713 GZ Groningen, Netherlands; 13 Department of Nuclear Medicine and Molecular, University Medical Center Groningen, Hanzeplein 1, P.O. Box 30.001, 9700 RB Groningen, Netherlands; 14 Center for Medical Physics and Biomedical Engineering, Medical University of Vienna, AKH-4L Währinger Gürtel 18-20, 1090 Vienna, Austria; 15 Abteilung für Präklinische Bildgebung und Radiopharmazie, University of Tübingen, Röntgenweg 13, 72026 Tübingen, Germany; 16 Department of Cardiovascular and Thoracic Radiology, Assistance Publique Hopitaux de Marseille; University of Aix-Marseille, 264 rue Saint Pierre, 13385 Marseille, France; 17 High-Field and Hybrid MR Imaging, University Hospital Essen, Hufelandstrasse 55, 45147 Essen, Germany; 18 Department of Nuclear Medicine and European Institute for Molecular Imaging (EIMI), University of Münster, Albert-Schweitzer-Campus 1, building A1, 48149 Münster, Germany; 19 Division of Nuclear Medicine, Department of Biomedical Imaging and Image-guided Therapy, Medical University Vienna, Währinger Gürtel 18-20, Floor 5L, 1090 Vienna, Austria; 20 European Society of Cardiovascular Radiology, Neutorgasse 9/2, 1010, Vienna, Austria; 21 European Association of Nuclear Medicine, Schmalzhofgasse 26, 1060, Vienna, Austria

**Keywords:** Cardiac PET/MRI, Cardiac MRI, Hybrid imaging, Cardiac imaging, FDG

## Abstract

**Abstract:**

Positron emission tomography (PET) and magnetic resonance imaging (MRI) have both been used for decades in cardiovascular imaging. Since 2010, hybrid PET/MRI using sequential and integrated scanner platforms has been available, with hybrid cardiac PET/MR imaging protocols increasingly incorporated into clinical workflows. Given the range of complementary information provided by each method, the use of hybrid PET/MRI may be justified and beneficial in particular clinical settings for the evaluation of different disease entities. In the present joint position statement, we critically review the role and value of integrated PET/MRI in cardiovascular imaging, provide a technical overview of cardiac PET/MRI and practical advice related to the cardiac PET/MRI workflow, identify cardiovascular applications that can potentially benefit from hybrid PET/MRI, and describe the needs for future development and research. In order to encourage its wide dissemination, this article is freely accessible on the European Radiology and European Journal of Hybrid Imaging web sites.

***Key Points*:**

*• Studies and case-reports indicate that PET/MRI is a feasible and robust technology.*

*• Promising fields of application include a variety of cardiac conditions.*

*• Larger studies are required to demonstrate its incremental and cost-effective value.*

*• The translation of novel radiopharmaceuticals and MR-sequences will provide exciting new opportunities.*

## Introduction

Positron emission tomography (PET) and magnetic resonance imaging (MRI) have both been used for decades in cardiovascular imaging. Since 2010, hybrid PET/MRI using sequential and integrated scanner platforms has been available, with hybrid cardiac PET/MR imaging protocols increasingly incorporated into clinical workflows [1].

Because of its robustness and broad range of imaging capabilities, cardiac MRI (CMR) has become a standard of reference for a variety of cardiovascular applications for different diseases, including the quantification of left and right ventricular dysfunction, the determination of global and regional wall motion abnormalities, and tissue characterisation (scar, fat, and oedema), as well as valve function. On the other hand, PET is superb at absolute quantification of myocardial perfusion and coronary flow reserve as well as visualisation and quantification of specific processes at the molecular level, such as metabolism, inflammation, or innervation [2]. In fact, there is a range of complementary information from each method, suggesting the use of hybrid PET/MRI may be justified in a routine clinical setting for evaluation of different disease entities [3].

However, there is also overlapping diagnostic information provided by both modalities, so that the combined use of the hybrid technique with regards to a potential incremental value for both diagnostic purposes and individual risk stratification of heart diseases has to be evaluated. For instance, both modalities have proven their ability to assess myocardial perfusion or viability, so that parameters coming from MRI or PET could be prioritised to avoid redundant information and to shorten acquisition protocols. Alternatively, specific imaging biomarkers could be added to increase the complementary multiparametric readout of the whole procedure. To elucidate the exact role and value of integrated PET/MRI in cardiovascular imaging, we aimed to give a technical overview of cardiac PET/MRI, provide practical advice related to the cardiac PET/MRI workflow, identify cardiovascular applications that can potentially benefit from hybrid PET/MRI, and describe the needs for future development and research. The content of this paper will be based on published studies and available evidence, and if not yet available, on personal experience and expert opinion.

## Technical considerations

### Design of current PET/MRI systems

The combination of separate PET and MR scanners into a single multimodality system necessitates a careful design of several hardware components. MRI requires well-controlled and uniform static magnetic fields for spin polarisation, linear gradient fields for spatial signal encoding, and radiofrequency (RF) fields for spin excitation and signal readout. Therefore, any unshielded hardware can affect the accuracy and quality of MR images. Furthermore, conventional PET detectors and associated hardware are not compatible with strong electromagnetic fields. In particular, conventional photomultiplier tubes (PMT), which are needed to convert and amplify signal from scintillation crystals into electronic signal, do not function properly in or near these fields.

Integrating PET detectors based on PMT within an MR system has proven to be a difficult task requiring some vendors to physically separate the PET and MRI units. In the context of hybrid PET/MRI, conventional PMT detectors can only be used if the PET unit is placed sufficiently far from the MRI unit to virtually eliminate deleterious interactions, thus leading to a sequential system design [4]. In current integrated PET/MRI systems interference is minimised using either avalanche photodiodes (APD; Siemens Biograph mMR) or silicon photon multipliers (SiPM; GE Signa PET/MR) [5–8]. The performance of these semiconductor-based detectors, used to replace conventional PMT, is not affected by strong magnetic fields, which is a precondition for the design of integrated PET/MRI systems.

### Attenuation correction (AC) in PET/MRI

Without provisions, PET systems miss certain coincidence events because of absorption in dense tissue or scattering out of the detector field of view (FOV). Thus, modern PET and PET/CT systems use attenuation maps (μ-maps) that contain the radiodensity of each voxel within the body volume for 511-keV photons for the correction of acquired PET data. These are typically calculated using transmission scans with external ^68^Ge radionuclide sources or now increasingly using co-registered CT data acquired at an energy level of 80-140 keV, which needs an additional extrapolation to convert to the radiodensity of 511-keV photons [9].

The practical consequence is that all PET data in hybrid PET/MRI needs to be attenuation corrected during the data reconstruction process in order to provide a valid quantification of regional tracer activity in the imaged object. Both patient tissues and PET/MRI system hardware components (e.g. the systems patient table, MR radiofrequency coils, ECG equipment) within the FOV of the PET detector during data acquisition attenuate the number of true annihilation events and, consequently, may lead to incorrect quantification results without applying attenuation correction (AC) [7, 10]. Depending on the relative position of the heart in the patient’s body, the emitted photons experience different attenuation on their way through the body to the PET detector. Thus, non-AC PET data show significant underestimation of the tracer activity deep in the patient’s body, including parts of the heart. Current system implementations for attenuation correction in integrated PET/MRI are realised in two ways: (1) The AC of patient tissues is performed by using MR-based image information that is segmented into different tissue classes and is assigned different linear attenuation coefficients (LAC). (2) The AC of hardware components is realised by using CT-based models that represent attenuating hardware (e.g. RF coils and the systems patient table) during the PET reconstruction [11].

### AC of patient tissues

**Fig. 1:**
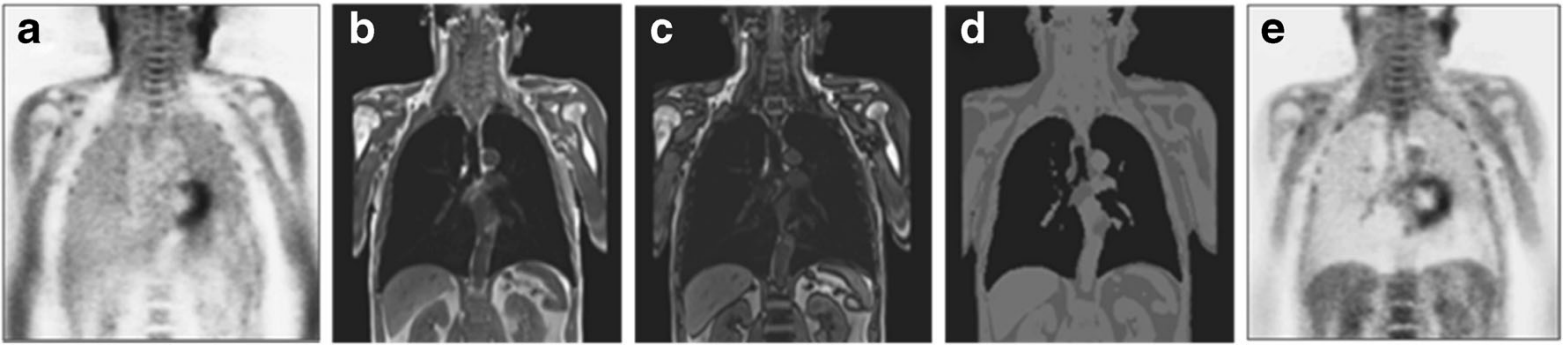
Soft-tissue attenuation correction (AC) of ^18^F-FDG PET images based on MRI. (A) Uncorrected thoracic PET scan showing relative activity enhancement in the lungs and along the outer contours of the patient. (B and C) 3D Dixon volume interpolated breath-hold examination (VIBE) MR sequence providing separate water/fat “in-phase” and “opposed-phase” images that serve as basis for soft-tissue segmentation. (D) Segmented soft tissue groups (air, fat, muscle, lungs) that can be assigned to a 3D PET attenuation map. (E) Resulting attenuation corrected PET scan of the initial data set (A). Note: Bone signal is assigned soft tissue values in this MRI-based approach for AC

In integrated PET/MRI, the PET AC factors need to be derived from the MRI data collected during a PET/MRI examination. The most common approach uses a multi-point Dixon sequence for segmentation into lung, fat, soft tissue, and background [11] (Fig. 1). While this method is widely used and has shown to provide robust results for MRI-based AC in whole-body PET/MRI, this method does not segment bone and calcification into a separate class, but classifies it as soft tissue, which may result in underestimation of local attenuation values close to bone. Consequently, standardised tracer uptake close to bone may be underestimated [12], which could in particular apply to the retrosternal parts of the heart.

Modified segmentation methods based on ultrashort echo time (UTE) sequences are now also available that can segment tissue with very short T2* (such as bone) into a separate class. The application of UTE sequences for bone detection has been shown to improve quantification in brain PET/MRI studies [13–16]. However, due to a rather small field of view and extended data acquisition times this technique is not yet usable in clinical cardiac PET/MRI imaging. For whole-body and cardiac PET/MRI imaging, the use of Dixon-based MR-AC [11] in conjunction with recently developed patient-specific atlas-based addition of major bones as a separate tissue class seems to be a practical alternative for further improvement of the current implementations of MR-based AC [17].

**Fig. 2:**
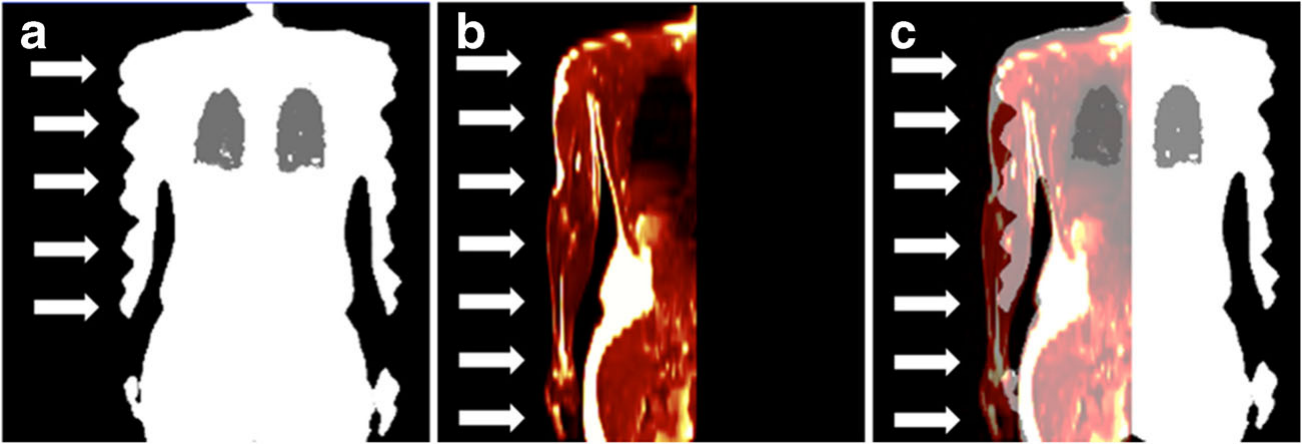
Truncation artefact correction in hybrid PET/MRI imaging. The FOV in MR imaging is limited, leading to signal truncations in the MR-based attenuation map (A, *arrows* along the arms). These signal truncations cause an attenuation bias during attenuation correction (under-correction). The HUGE technique increases the FOV in MR imaging eliminating these artefacts (B, *arrows*). The fusion of truncated attenuation map (A) and the HUGE images (B) shows this effect (C)

Because of lack of space within the magnet bore and relatively long examination times, cardiac PET/MRI is usually performed with the patient’s arms aligned along the body axis. Depending on the patient’s body habitus, this can result in parts of the arms being placed outside the MR FOV. This can cause so-called truncation artefacts, which can at least partially be corrected with PET emission data using maximum likelihood reconstruction of attenuation and activity (MLAA) [18]. The MLAA algorithm detects the contours of the arms in the PET images utilising unspecific radiotracer uptake, which allows the MR-based AC map to be complemented with PET information. This method is, however, limited when highly specific tracers are used which do not accumulate over time in non-target tissue such as the arms. An alternative method for truncation correction in MR-based AC has been proposed by Blumhagen et al. Here the transaxial FOV of the AC map is increased from typically 50 cm to 60 cm by applying optimised readout gradients in MR imaging (B0 homogenisation using gradient enhancement, HUGE) [19]. This method, based purely on MR information, effectively helps to reduce the residual attenuation bias in the thoracic region that might arise due to truncation artefacts along the arms [20, 21] (Fig. 2).

### AC of hardware components

**Fig. 3:**
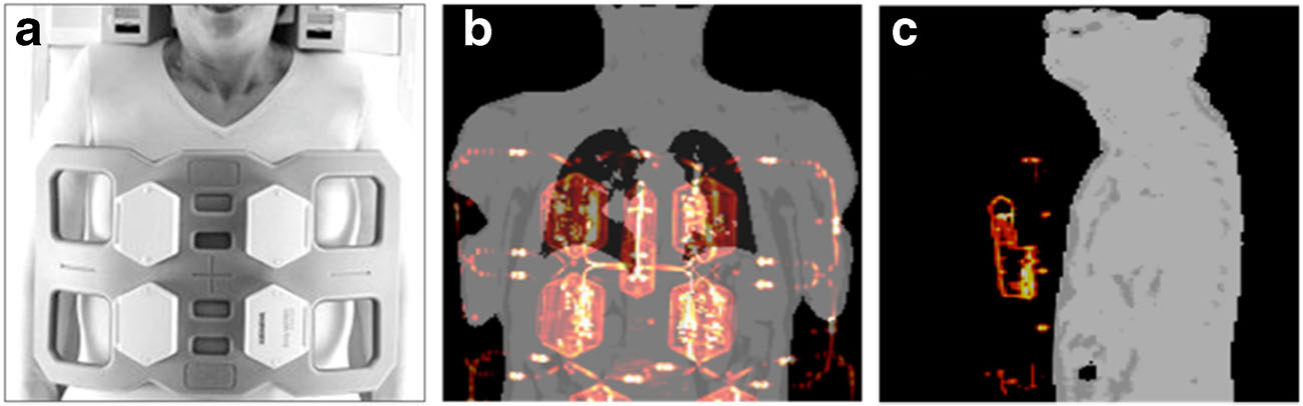
(A) 6-channel thorax radiofrequency coil that can be used for MR signal reception during simultaneous cardiac PET/MR data acquisition. Images (B and C) show MR-based attenuation maps of the patient tissues that were acquired with a 3D Dixon sequence. The hardware attenuation correction map of the flexible RF coil (*orange/red*) here was automatically co-registered with non-rigid registration to the patient tissue AC map using visible markers. Such attenuation maps represent the geometric distribution of PET signal attenuating hardware and soft tissue structures in the PET field of view during simultaneous PET and MR data acquisition

In integrated cardiac PET/MRI, the patient is placed on top of a rigid phased-array spine RF coil. For anterior signal detection, a second body phased-array RF surface coil is placed on the chest (Fig. 3). As attenuation of such instrumentation cannot be derived from the available MRI data, all RF surface coils used in the PET FOV during simultaneous PET data acquisition have to be optimised for PET transparency [22–24].

The residual PET signal attenuation of rigid and stationary equipment such as RF coils can be compensated by straightforward AC methods. Predefined attenuation maps (templates) for the patient table and non-flexible RF coils thus are usually added to the patient tissue attenuation map prior to the PET reconstruction. These templates are based on CT transmission scans of the patient table or RF coils, providing an exact 3D representation of the spatial distribution of attenuation factors in a virtual model of the respective hardware component [24, 25]. By linking the current patient table position during a patient examination to the known position of the hardware component on the table, template-based AC can be automatically performed during the PET data reconstruction process [7, 22]. This method for hardware component AC is an established standard in the current commercially available PET/MRI systems.

For flexible surface RF coils such as the 6-channel RF body phased array (Fig. 3), the AC must be performed differently. Because the design is flexible, the individual position and shape of this RF coil during a patient examination is not known. Thus, a pre-acquired rigid 3D CT template cannot directly be co-registered [24]. Here, MR-detectable markers can be used to perform an automatic non-rigid co-registration of the pre-acquired 3D CT attenuation template to the individual position and shape of the flexible RF during the cardiac PET/MRI examination [26]. Coil position detection with application of UTE sequences as an MR-based alternative has turned out to be insufficient for large flexible RF coils [24].

The previous sections on attenuation correction illustrate the complexity of performing AC in PET/MR imaging. In the lack of a radioactive transmission source as, for example, the CT scanner in PET/CT, or rotating ^68^Ge rod sources in former PET-only systems, new methods had to be developed to perform AC in the PET/MR environment [7]. As described in the previous sections, AC in PET/MR is assembled from many modular building blocks: Dixon sequences provide AC of the patient soft tissues [11], UTE sequences [15, 16] or bone models [17] provide AC of the major bones, pre-acquired CT-based templates provide AC of RF coils and other hardware components [22–26], truncation correction may be based on MR images (e.g. HUGE method) [19, 20] or based on PET images (e.g. MLAA method) [18]. Numerous innovative solutions for attenuation correction and truncation correction have been suggested and scientifically evaluated during the past years. Of these, some of the most accurate and practical developments have found their way from research applications into the most recent product software applications of all PET/MR systems. Today, attenuation correction in PET/MR whole-body imaging applications can be considered as largely solved in terms of robustness and accuracy. Nevertheless, the modular setting requiring the combination of multiple methods to achieve AC in PET/MR renders the situation comparably complex when compared to the “push-button” ease of use of performing AC in PET/CT.

### Motion correction

MR-based correction of motion in PET data is a further theoretical advantage of simultaneous PET/MRI acquisition, but it is still a highly promising topic of ongoing research and, thus, not yet implemented in clinical routine. In principle, the detection of respiratory and cardiac motion as well as involuntary patient movement using real-time 3D MRI and/or tagging techniques can be used to compensate for such motion in simultaneously acquired PET data [27]. Similarly, MRI-recorded 3D motion correction can be used for improved PET AC, allowing counts to be shifted before reconstruction [28, 29], increasing the reliability of the PET data as compared to self-gated methods, especially in low-dose/high-noise data [30, 31]. Once clinically available, these techniques would however require integrated scanners with the capacity of simultaneous image acquisition.

### Image post-processing, visualisation, and quantification

Several software solutions, available from commercial and academic sources, offer semi-automatic and automated processing of cardiac imaging data. Most software products on the market focus on either CMR or PET separately, but there are increasingly software packages such as OsiriX (OsiriX Foundation, Geneva, Switzerland), Munich-Heart (TU München, Munich, Germany), and *syngo*.via (Siemens Healthineers, Erlangen, Germany) available utilising both PET and MRI data. However, software solutions for fully integrated PET/MRI analyses are still missing. The following sections provide an overview of potential clinical indications for cardiac PET/MRI, which are also summarised in Table 1.

**Table 1:** Overview of potential clinical indications for cardiac PET/MRI

Potential	Indication	Comment
Strong	Cardiac inflammation	Detection and assessment of activity not satisfactory using CMR; complementary information using CMR and PET; high number of clinical cases
Strong	Ischemic heart disease	Established criteria for clinical outcome after revascularisation not satisfactory; integration of structural alterations and perfusion using PET/MRI seems plausible; high number of clinical cases with large combined morbidity and mortality; to date highly speculative due to missing data, particularly on the combination with MR coronary angiography.
Strong	Ischemic cardiomyopathy	Limited evidence available for combined PET/MRI, but generally great potential by assessing perfusion, metabolism, viability and function simultaneously. Strong potential for tissue characterization by using novel radiopharmaceuticals.
Intermediate	Acute coronary syndromes	Assessment of myocardial salvage and cardiac remodelling could guide the development of novel therapies; maybe limited relevance outside research or clinical studies
Weak	Cardiac tumours	CMR or PET(/CT) probably sufficient in most cases; anticipated added value of PET/MRI for planning of complex surgery/radiation or identification of relapse in malignant tumours

## Ischaemic heart disease

### Stable coronary artery disease

Stable coronary artery disease is among the major public health issues of Western societies and has traditionally been perceived as a consequence of the increasing narrowing of the coronary due to accumulation of atherosclerosis that can be diagnosed by coronary angiography and treated by percutaneous or surgical coronary revascularisation [32]. However, in contrast to the poor prognostic value of the morphologic severity of epicardial stenoses only the evidence of the haemodynamic relevance of coronary stenoses and/or the presence and extent of myocardial perfusion defect represent an established criterion for clinical outcome after revascularisation [33]. Despite some promising recent developments in self-navigated acquisition techniques [34] the detection and characterisation of coronary stenoses, remains the cornerstone of computed tomography [32]; however, myocardial perfusion imaging by MRI has been well established in clinical workflows and provides similar diagnostic accuracy as SPECT [35]. Also, PET is used for myocardial perfusion and in fact is widely considered the reference standard for non-invasive quantitative assessment of myocardial perfusion [36, 88]. Most of the myocardial perfusion tracers used in PET are produced onsite in cyclotrons, e.g. NH_3_ or H_2_^15^O, and have been extensively used in both research and clinical applications. With introduction of generator-based ^82^Rb PET based myocardial perfusion assessment is now also possible without an onsite cyclotron. With ^82^Rb the radiation dose is low and the spatial resolution seems to be sufficient for routine applications and has several advantages compared to SPECT-based myocardial perfusion [37, 38]. However, current design does not allow an easy workflow for ^82^Rb on a PET/MRI system as the generators are not safe to be operated within a strong magnetic field, novel ^18^F-labelled perfusion tracers with a 110-min half-life, high first-pass extraction, and nearly linear flow-related uptake were introduced [39] and quantitative assessment of the transmural extension of perfusion defects has been demonstrated [40]. However, these tracers are not yet broadly available. Despite the promise of PET/MRI hybrid systems, there is currently no scientific evidence to support the value of a combined PET/MRI approach for patients with stable coronary artery disease, thus further research is warranted [41].

### Acute coronary syndrome

Infarct size is a major predictor of outcome early after myocardial infarction. Initial PET/MRI studies in the subacute phase after acute myocardial infarction (AMI) have demonstrated moderate to good agreement between myocardial segments showing LGE and reduced ^18^F-FDG uptake [42–44]. Furthermore, MRI in the subacute phase after reperfused myocardial infarction is able to differentiate between myocardial oedema, microvascular obstruction, and intramyocardial haemorrhage as a potential indicator for reperfusion injury with additional prognostic impact also for the right ventricle [45–47].

Nevertheless, studies have observed a certain disagreement between PET and MRI. In cases of relative underestimation of infarct size using PET, this could be explained by the higher spatial resolution of MRI and the resulting detection of subendocardial infarction or a relative overestimation of infarct size using CMR, as gadolinium has been shown to be also entrapped in the surrounding oedema during the subacute phase of myocardial infarction [48]. However, studies have identified cases where some myocardial segments demonstrated reduced ^18^F-FDG uptake in PET, but no LGE in MRI. These myocardial segments had wall motion abnormalities and showed only partial functional recovery after 6 months [44].

The discrimination between reversible and irreversible myocardial dysfunction in the subacute phase after AMI is of high clinical and scientific importance. From the clinical perspective, reversible dysfunction of myocardial segments will contribute to global left ventricular recovery. On the other hand, dysfunction of non-infarcted myocardial segments in peri-infarct regions is linked to the salvage area, that is, the difference between the area at risk and the final infarct size. A PET/MRI study in patients with reperfused AMI has found that the area of reduced ^18^F-FDG uptake correlates with the area at risk (as determined by the endocardial surface area) and, in the absence of necrosis, is localised in the perfusion territory of the culprit artery [42]. If confirmed by further studies, the area of reduced ^18^F-FDG uptake could be used as a surrogate parameter for the evaluation of strategies to reduce infarct size, such as pre- and post- or remote conditioning.

Finally, with the ability of hyperpolarised compounds, e.g. ^13^C-pyruvate, to reveal in real time further details on myocardial substrate metabolism [49], and the recent demonstration of the feasibility of performing simultaneous PET and hyperpolarised MR, a technique named hyperPET [50], cardiac hyperPET on PET/MRI hybrid systems may become a future application for integrated scanners.

### Ischaemic cardiomyopathy

**Fig. 4:**
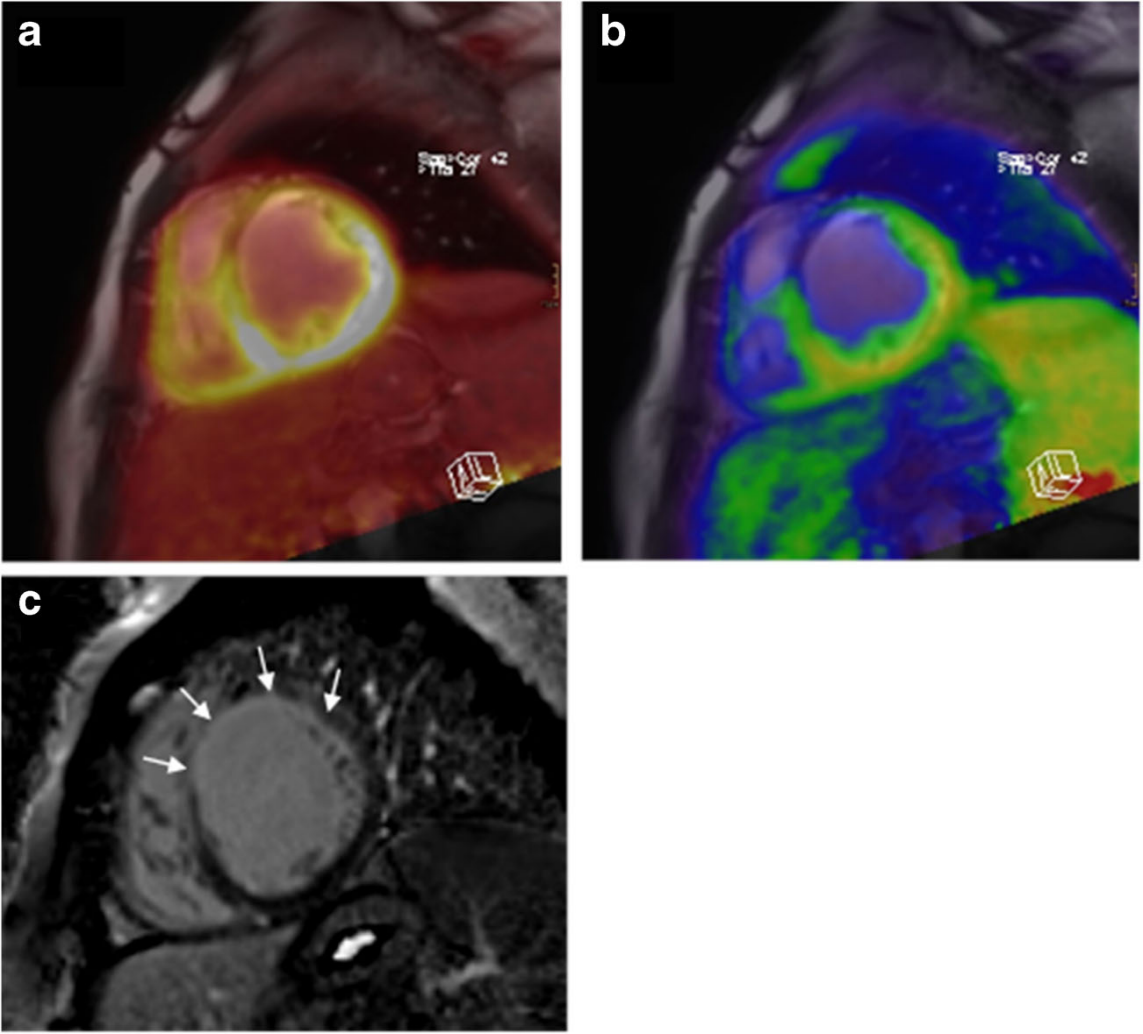
Cardiac PET/MRI of a 57-year-old male patient with advanced coronary artery disease and deteriorated LV function showing an agreement of missing ^18^F-FDG and ^13^N-NH3 uptakes with presence of transmural late gadolinium enhancement in the anterior wall of the myocardium, which was rated as transmural scar. (A) Image fusion of ^18^F-FDG PET and LGE MRI. (B) Image fusion of ^13^N-NH3 PET and LGE MRI. (C) Extent and transmurality of scar determined by MRI LGE

In patients with ischaemic cardiomyopathy, assessment of myocardial viability and function is of paramount importance for optimal patient management and further therapy planning. A wide range of treatment options are available including conservative medical treatment or revascularisation procedures. The role of cardiac PET/MRI in these patients could be the prospective identification of those patients who will eventually benefit from revascularisation therapy, as these invasive procedures are associated with significant periprocedural morbidity and mortality [51] and, thus, could be particularly useful in patients with reduced general condition due to congestive heart failure. For acquiring different blood flow and viability parameters in one session, integrated cardiac PET/MRI using dual tracer protocols such as ^13^N-NH_3_ and ^18^F-FDG combined with late gadolinium enhancement (LGE) can be applied. These allow for accurate quantification, localisation, and characterisation of the whole myocardium. While LGE enables precise assessment of transmural and even thin subendocardial scarring, simultaneous acquisition of sequential ^13^N-NH_3_ and ^18^F-FDG PET scans can identify and quantify myocardial areas with decreased perfusion or contractility, but preserved viability, so called hibernating or stunned myocardial tissue [52]. As LGE and ^13^N-NH_3_/^18^F-FDG provide different information, the interaction and complementary value of both techniques could improve both individual risk assessment and prediction of patient outcome (Fig. 4). In order to shorten imaging protocols and increase efficiency, future studies will have to identify complementary and redundant information yielded by the different techniques. Once the optimal PET/MRI viability parameters are identified, redundant MRI sequences and/or PET tracers could be omitted from the protocol. Also, since ^13^N-NH_3_ needs an onsite cyclotron and is labour intensive to produce, the use of other PET perfusions tracers for such combined studies could be considered.

Since patients with ischaemic cardiomyopathy are at high risk for developing ventricular arrhythmias and sudden cardiac death, a PET tracer of myocardial innervation could be added to the imaging protocol in these patients. Harms et al. have shown in a very recent publication that myocardial blood flow and myocardial innervation can be obtained from a single PET scan using ^15^O-water and ^11^C-meta-hydroxyephedrine [53]. Myocardial perfusion-innervation mismatches are frequently associated with increased mortality due to sudden cardiac arrest [54]. Prospective studies with combined PET/MRI and innervation tracers such as ^11^C-meta-hydroxyephedrine might, therefore, be usable to depict cardiac sympathetic neuronal dysfunction and to deliver comprehensive information about the denervated myocardium in patients with ischaemic cardiomyopathy, which are at increased risk of sudden cardiac death.

The main limitation of the current PET/MRI studies using dual tracer protocols is the long scanning time of 60 min or more, which may not be tolerated well by a subgroup of patients with poor general state. Thus, the development of shortened acquisition protocols remains a major goal of future research.

## Inflammatory heart disease

In recent years, ^18^F-FDG PET has attracted growing interest in the diagnosis and monitoring of inflammatory diseases, including those of the heart [55]. CMR is an established component in the clinical diagnosis and management of cardiac inflammation [56–58]. With MRI it is possible to detect even small areas of myocardial necrosis or fibrosis using LGE and T1 mapping, to accurately detect regional and global dysfunction, to assess myocardial oedema and hyperaemia, as well as pericardial effusion. Despite the availability of newer T1 and T2 mapping techniques together with extracellular volume (ECV) calculations to detect also diffuse inflammatory disease, there is still a lack of accuracy especially in patients with chronic myocarditis [59]. However, in these patients with chronic disease, specific MR-inflammation parameters such as T2 mapping performed best. Given the specific characteristics of MRI, a combination of multiparametric MRI with the high sensitivity and outstanding quantification capabilities of ^18^F-FDG PET could represent a powerful imaging modality for cardiac inflammation. Furthermore, a number of non-FDG tracers targeting different aspects of inflammation reaching from chemokine expression over cellular involvement to changes in the myocardial tissue composition could add significant value to cardiac PET/MRI in the future [60]. For ^18^F-FDG PET imaging of myocardial inflammation it is important to realise, that healthy myocardium utilises both glucose and free fatty acids, depending on the patient’s nutrition and fasting status. To achieve high contrast between inflammatory infiltrates and normal myocardium, it is necessary to suppress myocardial glucose metabolism, which can be obtained by different protocols including prolonged fasting, high-fat low-carbohydrate diet, fatty acid loading, and unfractionated heparin loading [61–63]. However, fasting has been identified as one major reason for patient discomfort, potentially contributing to increased cancellation rates during cardiac PET/MRI examinations. A recent study has demonstrated a high-fat low-carbohydrate protein-permitted diet without fasting to yield an 84% success rate regarding suppression of normal myocardial glucose uptake. Cancellation rate was less than 3% and thus comparable to routine CMR examinations [64]. Novel techniques such as ultrasmall superparamagnetic particles of iron oxide (USPIO)-enhanced MRI targeting macrophage activity or ^68^Ga-labelled somatostatin receptor PET imaging targeting activated lymphocytes hold the potential to further improve cardiac PET/MR imaging of inflammation.

### Myocarditis

**Fig. 5:**
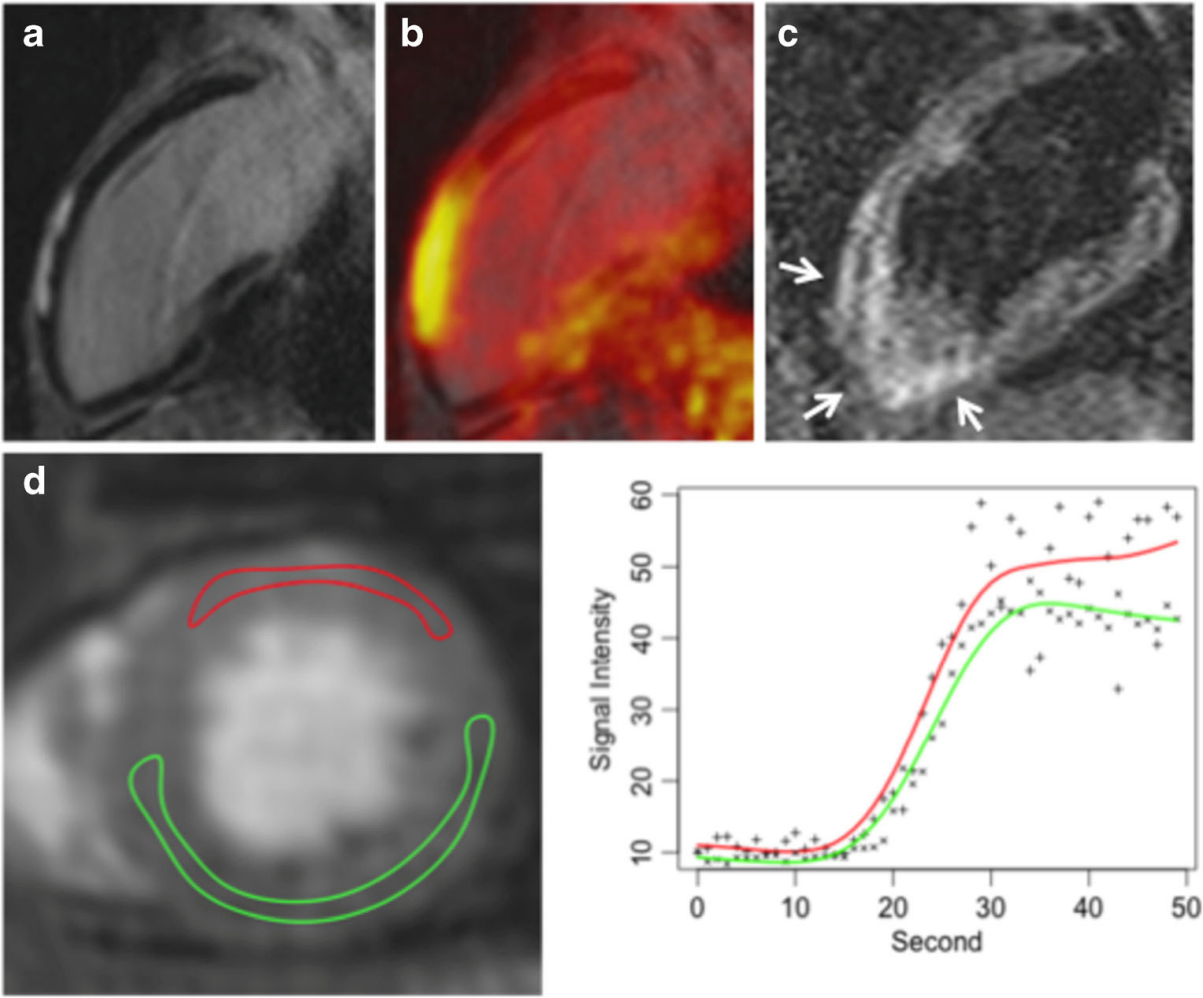
^18^F-FDG PET/MRI in a patient with acute viral myocarditis caused by parvovirus B19. (A) Late-gadolinium-enhanced MRI long-axis view demonstrating typical subepicardial enhancement in the anterior left ventricular wall that was in excellent spatial agreement with increased ^18^F-FDG uptake on fused images (B). (C) T2-weighted images revealed an oedema in the LV anterior wall. (D) Dynamic perfusion imaging revealed hyperaemia in the LV anterior wall. (With kind permission from Ref 50-Nensa, Poeppel 2014)

To date, no studies but some case reports have been published that evaluate PET/MRI for the imaging of myocarditis. An initial report has demonstrated the use of ^18^F-FDG PET /MRI in a case of myocarditis caused by parvovirus B19 [65], Figure 5. In this report, focal subepicardial LGE was closely matched by intense ^18^F-FDG uptake and accompanied by myocardial oedema and hyperaemia. While MRI would have been sufficient for the detection and diagnosis of myocarditis in this case, it highlights the ability of PET to quantify inflammatory activity, particularly with respect to disease monitoring. A similar case was reported from a patient with myocarditis due to Epstein-Barr virus infection with diffuse ^18^F-FDG uptake in the lateral wall that again closely matched LGE and myocardial oedema [66]. Besides monitoring of disease activity, integrated assessment using PET/MRI could increase diagnostic accuracy in cases with ambiguous MRI findings or improve differentiation between acute and chronic/persistent myocarditis. Inflammatory cells can utilise a high amount of glucose and, thus, might express high levels of glucose transporters and increase hexokinase activity, leading to increased ^18^F-FDG uptake in inflammatory infiltrates. As such, ^18^F-FDG uptake is truly complementary to LGE (necrosis and oedema), T2-weighted MRI (oedema) and early gadolinium enhancement (hyperaemia) and could be useful to extend the well-known “Lake Louise Criteria” in the non-invasive assessment of myocardial inflammation in patients with suspected myocarditis [67]. Despite the fact that myocarditis and pericarditis often co-occur as so-called perimyocarditis, to date no studies or reports on the use of PET/MRI in pericarditis have been published. Still it seems plausible, that the detection and quantification of active inflammation using PET can here be considered to be as much an opportunity as in myocarditis.

### Cardiac sarcoidosis

Cardiac involvement is a strong predictor of poor outcome in sarcoidosis, often manifesting in arrhythmia and heart failure. Consequently, early detection of this complication—including the differentiation between active and chronic disease—is a prerequisite for early treatment and thus can contribute to the reduction of overall morbidity and mortality. Commonly, treatment not only comprises symptomatic management of cardiac dysfunction, but also immunosuppressive therapy, which needs to be carefully balanced against potential side effects. Thus, besides early diagnosis, also continuous monitoring of disease activity with the objective of drug titration is needed. However, both accurate detection and monitoring of cardiac sarcoidosis (CS) remain challenging, especially as endomyocardial biopsy significantly suffers from sampling error and can cause severe complications such as myocardial perforation. Hence, non-invasive techniques such as CMR and ^18^F-FDG PET represent interesting alternatives in the clinical workup of this disease [67]. In fact, MRI and ^18^F-FDG PET both are recommended for the assessment of CS [68]. CMR has been demonstrated to predict death and other adverse events in suspected CS [69] and a meta-analysis (seven studies, 164 patients) reported a pooled 89% sensitivity and 78% specificity for ^18^F-FDG PET in the detection of cardiac involvement in sarcoidosis [70]. In a comparative study of CMR and ^18^F- FDG PET, MRI provided a higher negative value and thus might be superior for ruling out cardiac involvement [71].

However, there is already evidence that a combination of both modalities might provide added value [75]. Both imaging modalities in fact visualise different pathologic correlates of CS. While LGE can accurately detect myocardial oedema necrosis and fibrotic scar, ^18^F-FDG uptake is a quantifiably surrogate parameter of increased glucose metabolism, a hallmark of inflammation. Thus, a combination of CMR and ^18^F-FDG PET has the potential to provide both accurate detection as well as assessment of disease activity. Several case reports have demonstrated the feasibility of integrated ^18^F-FDG PET/MRI in the detection [73, 74] and therapy monitoring [76] of CS. One study in 51 consecutive patients with CS has found improved diagnostic accuracy of combined ^18^F-FDG PET/MRI over PET and MRI alone [72]. Despite the sparseness of available studies, integrated ^18^F-FDG PET/MRI holds great promise in the imaging of CS. However, further evaluation, particularly with respect to clinical outcome of patients is warranted.

### Endocarditis

Endocarditis is a common complication after prosthetic valve implantation. Complications of active inflammation include dehiscence of the valve, paravalvular leaks and abscesses. MRI allows direct visualisation of the valve, identification and quantification of regurgitations, and may spot separation of the aortic valve from the aortic annulus [77]. Of note, artefacts arising particularly from mechanical valves may have a severe impact on image quality and diagnostic accuracy. In contrast to MRI, ^18^F-FDG PET allows the direct assessment of the predominant site and acuity of inflammation and may also be utilised to monitor therapy response after start of antimicrobial treatment. An important prerequisite to increase specificity of ^18^F-FDG PET is the effective suppression of physiologic, myocardial ^18^F-FDG uptake. Different patient preparation protocols (including prolonged fasting, high-fat low-carbohydrate diet and pre-injection of heparin before ^18^F-FDG administration) have been proposed [78]. Furthermore, review of non-attenuated PET images is obligatory as metal extinction artefacts on MRI translate into the attenuation correction map and cause underestimation of the ^18^F-FDG uptake on attenuation-corrected PET images (in contrast to metal artefacts in CT, which cause an overestimation of tracer uptake on attenuation-corrected PET images) [79]. So far, to the best of our knowledge, no literature exists on the use of integrated PET/MRI in infectious endocarditis. One case report describes the successful use of ^18^F-FDG PET/MRI in a patient suffering from Loeffler endocarditis [80].

## Cardiac tumours

**Fig. 6:**
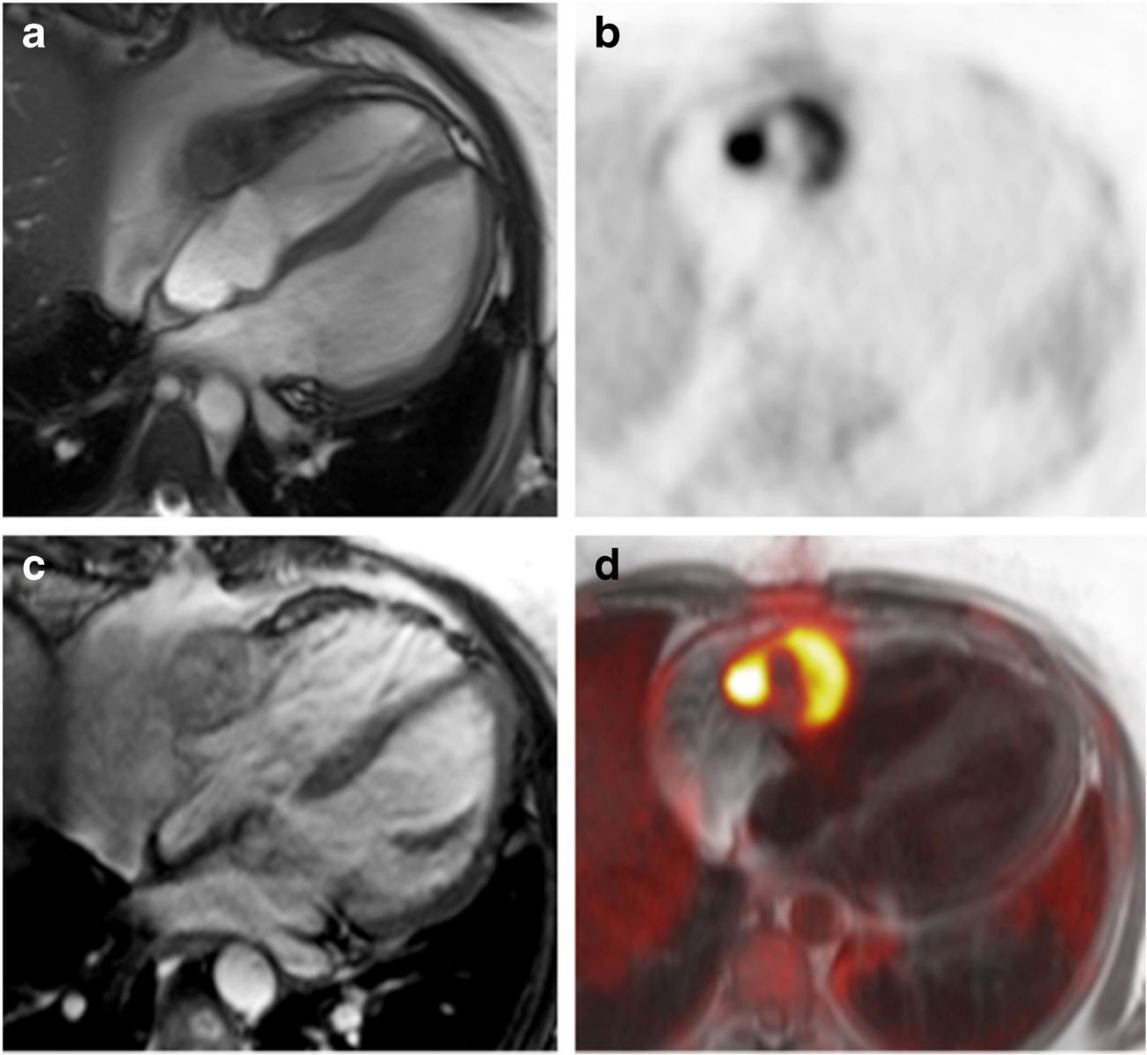
(A) Cine MR image of an angiosarcoma infiltrating the free wall of the right ventricle and atrium with adjacent pericardial effusion. (B) ^18^F-FDG PET shows intense but heterogeneous ^18^F-FDG uptake within the tumour and otherwise suppressed myocardial ^18^F-FDG uptake by the use of a high-fat low-carbohydrate protein-permitted diet. (C) The tumour demonstrates heterogeneous and overall moderate enhancement on T1-weighted MRI after intravenous application of gadolinium-based contrast agent. (D) Fused images show excellent spatial agreement between PET and MRI

Given the role of MRI in the assessment of cardiac tumours and the overall power of ^18^F-FDG PET in oncologic imaging, a combination of both seems to be an attractive combination for the diagnostic workup of cardiac masses (Fig. 6). However, both modalities alone have already been demonstrated to yield high diagnostic accuracy in the assessment of malignancy [81], which is one of the most important clinical questions for non-invasive assessment before treatment. Nevertheless, integrated assessment yielded improved diagnostic accuracy over PET- or MR-only assessment in a small pilot study including 20 patients with cardiac masses [82]. CMR has already been proven beneficial in discriminating brown fat from pericardial metastasis using its well-known tissue characterisation capabilities [76]. A recent JACC imaging vignette shows the potential of PET/MRI in the diagnosis of cardiac and paracardiac masses with histopathologic correlation [83]. Considering, the already strong diagnostic performance of PET/CT and MRI, high cost, and limited availability of PET/MRI scanners, integrated PET/MRI imaging might be reserved for selected cases of cardiac tumours were true benefit can be expected. Such cases could include the planning of surgery in patients with complex cardiac infiltration or the differentiation of scar tissue vs. relapse in follow-up examinations after surgery or radiation therapy [84]. However, further evaluation with respect to costs and radiation exposure is required.

## Radiation exposure

The effective radiation dose for patients undergoing any of the aforementioned procedures using standard PET hardware (normally PET/CT) ranges between 1 and 7 mSv. However, acquisition times of the MRI component are comparatively long. Depending on the half-life of the respective radiotracer, PET acquisition times exceeding twice the usual acquisition time without extension of the overall scan time are possible for PET/MRI. ^18^F has a half-life of 110 minutes. Thus, a significant reduction in the administered activity (e.g. half of the usual activity) seems prudent [85]. As a result, the effective radiation doses are reduced by the same value. High-definition hybrid cardiac ^18^F-FDG PET/MRI has been shown to be diagnostic using a mean activity of 150±70 MBq [64]. Compared to PET/CT, PET/MRI allows for further radiation dose reduction due to the use of MRI data for attenuation correction, which has been demonstrated to be robust with an absolute mean difference of less than 2% for fat, muscles, and blood [86]. Furthermore, great advances have been made in PET hardware using APDs and SiPMs with improved image resolution, higher count rate, and reduced sensitivity to electromagnetic fields [87], introducing additional radiation dose reduction possibilities for PET/MRI. Doses go below doses that are common in CT angiography and compete with what is possible with the latest technology on dual source CT using prospectively triggered high-pitch spiral acquisition [88].

## Pitfalls

Initial experience after approximately 7 years and several hundred examinations applied at various sites worldwide has demonstrated that integrated cardiac PET/MRI, although a complex procedure, is a robust and reliable imaging modality feasible for clinical routine diagnostics. However, we still recommend that a team of MRI and PET cardiac imaging specialists and technicians perform and interpret cardiac PET/MRI, as basic knowledge about common pitfalls is crucial for valid image interpretation.

### Segmentation and misalignment errors

As the creation of μ-maps is based on segmentation of MR data, the validity of attenuation-corrected PET data is directly dependent on the validity of the underlying segmentation. MR image artefacts or unexpected behaviour of the segmentation algorithm can cause more or less severe tissue misclassification, compromising the validity of attenuation-corrected PET data due to wrong attenuation coefficients in the μ-map [89]. MR artefacts in cardiac imaging frequently originate from foreign objects such as implantable port systems, sternal wire cerclages, artificial heart valves or artificial joint replacement of the humerus. It is mandatory, that cardiac PET/MRI reading includes visual inspection of the underlying μ-maps. If significant errors are evident, findings in attenuation-corrected PET data should be interpreted with caution and correlation with uncorrected PET data should be performed.

Another typical AC-related issue results from patient motion. In a typical setting, the MR-based μ-maps are created in the beginning of the study and are later used for the attenuation correction of PET data that get continuously acquired over time in list mode. If the patient significantly changes body position following μ-map creation, this results in misalignment between attenuation coefficients and PET data, which can cause severe PET image artefacts and quantification bias. As a simple workaround it is recommended to perform repeated μ-map creation, interleaved between main MRI blocks. If patient motion is retrospectively detected, PET and MR needs to be registered and the PET reconstruction to be repeated. Alternatively, PET list-mode data can be truncated to an interval before or after patient motion and an appropriate μ-map can be selected for attenuation correction.

### Patient preparation for ^18^F-FDG PET/MRI studies

**Fig. 7:**
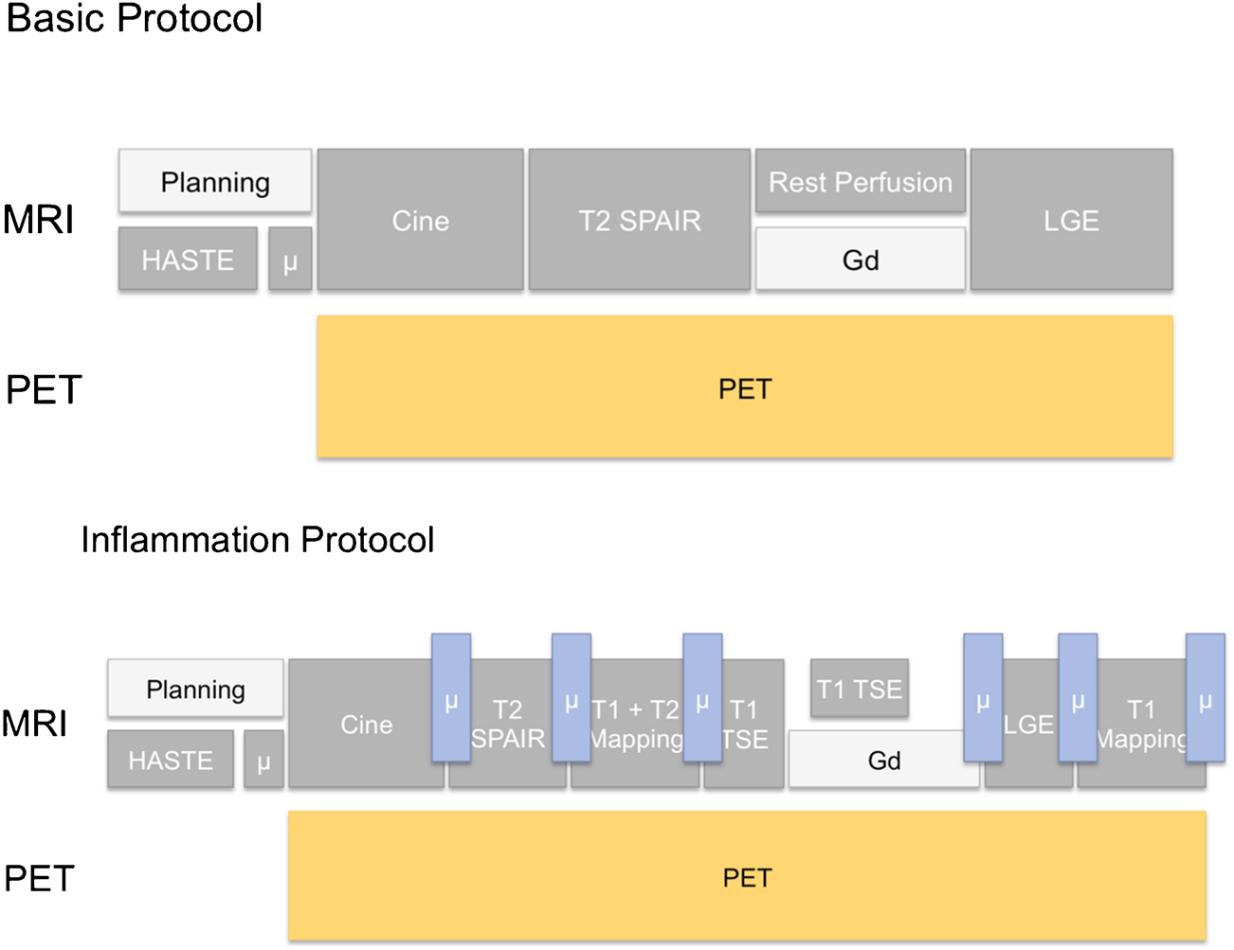
Sample basic and inflammation focused imaging protocol for cardiac PET/MRI

Depending on the clinical question, myocardial metabolism needs to be shifted towards free fatty acid or glucose utilisation in cardiac PET imaging with ^18^F-FDG; thus, reliable patient preparation is of utmost importance [90]. It is strongly recommended to perform detailed patient interviews regarding the compliance to the preparation protocol before tracer injection. In cases of incompliance, the PET/MRI examination can be postponed or certain countermeasures (e.g. insulin injection, unfractionated heparin injection, fatty acid loading) can be taken. In addition to interviews, it is recommended to perform blood testing of glucose level. A sample basic and inflammation focused imaging protocol for cardiac PET/MRI is provided in Fig. 7.

### Reporting

The procedure and the results of the cardiac PET/MRI study should be documented in a consensus report by respective PET and MRI specialists. The description of findings should be performed in a combined fashion rather than as a separate description of PET and MRI findings split in two sections. For performing and reporting of both cardiac PET and MRI we refer to the actual procedure guidelines [91–93]. Here we provide a brief summary with a special focus on integrated PET/MRI. We recommend reporting four key elements in the consensus report: (a) clinical information, (b) procedure description, (c) description of findings, and (d) summary.

#### Clinical information

The clinical information should include patient demographics (age, gender, weight, height) and medical history of the patient (known cardiac and extracardiac diseases, clinical symptoms, cardiovascular risk factors and history of prior events and treatments). It should also describe the indication for the study and the specific question of the referring physician.

#### Procedure description

Specific patient preparation (e.g. fasting or other dietary precautions, glucose loading, unfractionated heparin loading) and blood glucose level before FDG administration should be documented as well as the name, dose and route of administration of regulated non-radioactive drugs (e.g. insulin adenosine) and contrast agents. Study-specific information should include the radiopharmaceutical, the amount of injected activity in MBq, the route of administration (intravenous) and the date and time of administration. Information about the PET/MRI system (type, manufacturer, field strength) and specific equipment information (e.g. MR surface coils) should be specified. A description of the procedure should include the time interval between administration of the tracer and the start time of the data acquisition, as well as a short description of the MRI protocol used. If appropriate, the type of stress protocol should be specified.

#### Description of findings

The location, extent and intensity of pathological tracer accumulation or pathological tracer reduction related to normal cardiac tissue should be reported with regard to the aforementioned references and the recommendations for standardised myocardial segmentation and nomenclature for tomographic imaging of the heart [93]. The intensity of pathological tracer accumulation or reduction could be weighted as mild, moderate or intense. Quantitative measures of tracer uptake could be provided (e.g. SUV/SUL, perfusion [mL/min/g], metabolic rate), especially if a comparison to follow-up examinations is intended. Depending on the clinical indication, the report should include data regarding perfusion/viability/extent and transmurality of scar tissue, left and/or right ventricular function analysis, analysis of regional wall motion and myocardial mass, as described in detail previously. It also may include ventricular dimensions, which correlate to dimensions as measured in echocardiography to improve comparability. Depending on the MRI protocol used, the report should give detailed information about the localisation and extent of structural abnormalities of the myocardium including oedema, perfusion deficits, infarcts, microvascular obstruction, haemorrhage, and fibrosis. The relationship of relevant findings on MRI to pathological tracer distribution should be reported. Findings should be at least weighted as concordant/matching or discordant.

Extracardiac findings (e.g. great vessels, mediastinum, lung) should be reported as well.

Depending on availability, findings should be interpreted in the context of other imaging examinations (e.g. CT, PET/CT, SPECT/CT), MRI, coronary angiography, ultrasound) and clinical data. Particularly, assessment of hibernating myocardium frequently requires prior myocardial perfusion SPECT or PET. Comparison with previous examinations should be part of the report.

If appropriate, confounding factors that might influence the sensitivity or specificity of the assessment should be mentioned, e.g. motion artefacts, susceptibility artefacts, undesired distribution of the tracer (such as ubiquitous high or low ^18^F-FDG uptake in the myocardium, depending on the aims of the examination).

#### Summary

The study should be identified as normal or abnormal. The specific questions asked of the referring physician should be directly addressed. If possible, a definite diagnosis should be stated. Joint approval from a radiologist and a nuclear medicine specialist is encouraged. Document the communication of urgent or emergency findings to referring physicians or their representative.

### Requirements for future clinical applications

PET/MRI shows high potential for innovation in cardiac imaging, providing comprehensive anatomical, pathomorphological, functional, and molecular information of the myocardium, which might lead to improved diagnosis and therapy monitoring of cardiac diseases. As a new addition to the palette of advanced imaging modalities, integrated PET/MRI systems need to be validated before a broader clinical application can be accomplished [93].

There are multiple other issues remaining to be resolved and the strengths of simultaneous image acquisition were not yet sufficiently exploited. Further improvement of MR-based attenuation- correction is needed to allow for more accurate PET assessment. This will promote applications that—such as quantitative myocardial perfusion imaging—require quantitative PET imaging. Also, partial volume and motion corrections have to be clinically implemented for more accurate, reproducible and sensitive PET quantification for conventional and also for novel molecular agents. This requires the setup of multiparametric imaging protocols and specialised software for the integrated reading of multiparametric PET/MRI studies.

In a second phase, efforts have been made to understand the interrelationship between different PET and MRI parameters with the aim to resign redundant information and to validate the incremental value of potentially complementary parameters. It might for example not be necessary to estimate stress/rest perfusion, scar tissue or LV functional values with both techniques, PET and MRI, simultaneously. However, prospective clinical trials, uni- or multi-centric, to demonstrate added value regarding diagnosis, monitoring and most importantly patient outcome of certain parameters in a clinical setting are still missing.

The next step could then be the optimisation of imaging protocols for the most important indications and the evaluation of multiparametric (integrated) PET/MR imaging against hard clinical endpoints. Also, new areas in disease assessment should be explored, including the possible application of PET/MRI in the detection of disease which could not be detected using PET or MRI separately, for example low signal to noise scans in chronic low-grade inflammation disease. In this respect, the opportunities of multiparametric imaging need to be translated into diagnostic benefit to justify the investments in this rather complex and expensive technology.

## Summary

Integrated PET/MRI is available since 2010 and several studies and case reports have demonstrated cardiac PET/MRI to be feasible and robust. MR image quality is not compromised by the PET component and MR-based attenuation correction provides sufficient accuracy for most clinical applications. PET performance is comparable to PET/CT in the majority of cases. Promising fields of application include suspected coronary artery disease, acute myocardial infarction, and heart failure, inflammatory heart diseases such as myocarditis and cardiac sarcoidosis, as well as cardiac tumours. However, larger studies will have to demonstrate added value in comparison with current standards of care. Further research, and not least, economical review will have to clarify where integrated scanners demonstrate a competitive advantage and where sequential PET and MR acquisition with post hoc image fusion with software is good enough. Ongoing technical improvements such as MR-based PET motion correction are anticipated and will lead to higher spatial and temporal resolution, enabling advanced applications such as imaging of coronary atherosclerosis. Finally, the translation of already existing PET tracers from preclinical imaging into the clinical routine will open up exciting new possibilities.
